# Comprehensive essentiality analysis of the *Mycobacterium kansasii* genome by saturation transposon mutagenesis and deep sequencing

**DOI:** 10.1128/mbio.00573-23

**Published:** 2023-06-23

**Authors:** Keith Levendosky, Niklas Janisch, Luis E. N. Quadri

**Affiliations:** 1 Department of Biology, Brooklyn College, City University of New York, Brooklyn, New York, USA; 2 Biology Program, Graduate Center, Biology Program, Graduate Center, City University of New York, New York, New York, USA; 3 Biochemistry Program, Graduate Center, City University of New York, New York, New York, USA; Weill Cornell Medicine, New York, New York, USA

**Keywords:** *Mycobacterium kansasii*, gene essentiality, transposon mutagenesis, TnSeq, nontuberculous mycobacteria, mycobacterial orthology analysis, comparative mycobacterial gene essentiality, mycobacterial comparative genomics, pRAW-like plasmid, plasmid-encoded ESX system, tuberculosis, mycobacterial drug targets, antitubercular target candidate, mycobacterial ESX secretion system, type VII secretion system, nucleoid-associated protein

## Abstract

*Mycobacterium kansasii* (*Mk*) is an opportunistic pathogen that is frequently isolated from urban water systems, posing a health risk to susceptible individuals. Despite its ability to cause tuberculosis-like pulmonary disease, very few studies have probed the genetics of this opportunistic pathogen. Here, we report a comprehensive essentiality analysis of the *Mk* genome. Deep sequencing of a high-density library of *Mk Himar1* transposon mutants revealed that 86.8% of the chromosomal thymine–adenine (TA) dinucleotide target sites were permissive to insertion, leaving 13.2% TA sites unoccupied. Our analysis identified 394 of the 5,350 annotated open reading frames (ORFs) as essential. The majority of these essential ORFs (84.8%) share essential mutual orthologs with *Mycobacterium tuberculosis* (*Mtb*). A comparative genomics analysis identified 139 *Mk* essential ORFs that share essential orthologs in four other species of mycobacteria. Thirteen *Mk* essential ORFs share orthologs in all four species that were identified as being not essential, while only two *Mk* essential ORFs are absent in all species compared. We used the essentiality data and a comparative genomics analysis reported here to highlight differences in essentiality between candidate *Mtb* drug targets and the corresponding *Mk* orthologs. Our findings suggest that the *Mk* genome encodes redundant or additional pathways that may confound validation of potential *Mtb* drugs and drug target candidates against the opportunistic pathogen. Additionally, we identified 57 intergenic regions containing four or more consecutive unoccupied TA sites. A disproportionally large number of these regions were located upstream of *pe*/*ppe* genes. Finally, we present an essentiality and orthology analysis of the *Mk* pRAW-like plasmid, pMK1248.

**IMPORTANCE:**

*Mk* is one of the most common nontuberculous mycobacterial pathogens associated with tuberculosis-like pulmonary disease. Drug resistance emergence is a threat to the control of *Mk* infections, which already requires long-term, multidrug courses. A comprehensive understanding of *Mk* biology is critical to facilitate the development of new and more efficacious therapeutics against *Mk*. We combined transposon-based mutagenesis with analysis of insertion site identification data to uncover genes and other genomic regions required for *Mk* growth. We also compared the gene essentiality data set of *Mk* to those available for several other mycobacteria. This analysis highlighted key similarities and differences in the biology of *Mk* compared to these other species. Altogether, the genome-wide essentiality information generated and the results of the cross-species comparative genomics analysis represent valuable resources to assist the process of identifying and prioritizing potential *Mk* drug target candidates and to guide future studies on *Mk* biology.

## INTRODUCTION

Nontuberculous mycobacteria (NTM) are a broad group of bacteria related to *Mycobacterium tuberculosis* (*Mtb*), the causative agent of tuberculosis (TB). Many species of NTM are clinically relevant as opportunistic pathogens and, unlike *Mtb*, these pathogens can persist in the environment (1, 2). Among the NTM most commonly isolated from patients is *Mycobacterium kansasii* (*Mk*), a slow-growing opportunistic pathogen that can cause tuberculosis-like pulmonary disease in individuals that are immunocompromised or have other conditions considered risk factors for opportunistic mycobacterial infections, including chronic obstructive pulmonary disease and malignancy (3 - 9).

Most *Mk* primary lung infections are likely acquired by exposure to aerosolized environmental bacteria. *Mk* is frequently isolated from tap water and other urban water systems (10 - 13), the hypothesized primary pathogen reservoirs generating aerosolized bacteria and presenting a constant health risk for those susceptible to infection. Person-to-person transmission has not been reported. However, some studies suggest that it might be possible, and the potential emergence of strains with epidemiologically meaningful person-to-person transmission capacity is a concern (14).

Paralleling the drug treatments against the lung disease caused by members of the *Mtb* complex, the standard treatments for *Mk* infection are multidrug regimens that are expensive, long-term, and often hampered by adverse side effects (15 - 19). These factors contribute to reduced treatment compliance, which along with the rise of *Mk* isolates resistant to the first-line antimycobacterial drug rifampin (20), lead to increased rates of treatment failure (16, 17). In all, there is a clear need for new and more effective antimicrobial drugs to combat *Mk* infection. This need is further underscored by the potential rise of multidrug-resistant *Mk* strains as a collateral outcome of the pervasive use of anti-TB drugs in high TB burden areas of the world.

Of note, although *Mk* is currently classified into seven subtypes (I–VII), several recent studies using sequencing- and transcriptome-based approaches have proposed reclassifying these subtypes as closely related species (21 - 23). Subtype I is by far the most prevalent in human isolates and retains the species name *Mycobacterium kansasii* in these reclassification proposals. Besides laying the foundation for updating the molecular taxonomy of *Mk*, these studies have highlighted the observation made previously that *Mk* shares many recognized virulence determinants with *Mtb* (24). Indeed, *Mk* is one of the NTM most closely related to the *Mtb* complex, and it has been suggested that *Mtb* evolved into an obligate pathogen from an *Mk*-like environmental opportunistic pathogen (25). These observations might lend support to the use of *Mk* as one of the model organisms to help expand our understanding of the evolution and biology of the *Mtb* complex.

Despite the clinical relevance of *Mk* as one of the most pathogenic NTM, its biology remains largely underexplored. In particular, there is a noticeable paucity of literature reporting direct genetic manipulation of *Mk* to study gene function (26 - 30). Encouragingly, the completion of the *Mk* genome sequence was reported in 2015 (24). Thus, some aspects of *Mk* gene function, including gene essentiality, may be inferred from bioinformatics and extrapolation of insights gained for *Mtb* (24, 25), the main reference for comparative genomics of mycobacterial pathogens. However, while highly useful surrogates in the absence of bona fide experimental data, *in silico* approaches do not provide a complete picture and can often lead to flawed conclusions. These are germane considerations given that the 6.55 Mb *Mk* genome (chromosome plus a pRAW-like plasmid, pMK12478) (24) is ~50% larger than that of *Mtb* (31).

Without a doubt, experimentation focused on *Mk* is clearly necessary. In particular, genome-wide studies aimed at dissecting *Mk* gene essentiality will significantly expand our understanding of *Mk* biology and bring to light new potential drug target candidates. With these considerations in mind, we recently investigated and validated the applicability of the ϕMycoMarT7 phage-based specialized transduction method for *Himar1* transposon (Tn) mutagenesis in *Mk* and provided proof-of-principle for its use in combination with Tn insertion sequencing (TnSeq) (26, 32). This earlier work set the stage for the use of a saturation Tn mutagenesis–TnSeq approach to study gene essentiality in *Mk*, as done in several other species of mycobacteria (33 - 36). In this study, we performed a large-scale TnSeq analysis of a high-density library of *Mk* Tn mutants to predict genetic determinants required for *in vitro* growth. We also used comparative genomics and available gene essentiality data for *Mtb* (a slow-growing obligate pathogen) and the NTM *Mycobacterium avium* subsp. *hominissuis* (*MAH*, a slow-growing opportunistic pathogen), *Mycobacterium abscessus* (*Mab*, a rapidly growing opportunistic pathogen), and *Mycobacterium smegmatis* (*Msm*, a nonpathogenic rapidly growing species) to illuminate similarities and differences between the biology of these mycobacteria. Our analysis reported herein provides insights into the biology of *Mk*, informs future directions to prioritize studies on potential drug target candidates and avenues to novel therapeutics against *Mk* infections, and highlights possible biological underpinnings of *Mk* drug susceptibility characteristics.

## RESULTS AND DISCUSSION

### Insertion statistics and essentiality status of TA sites

**Fig 1:**
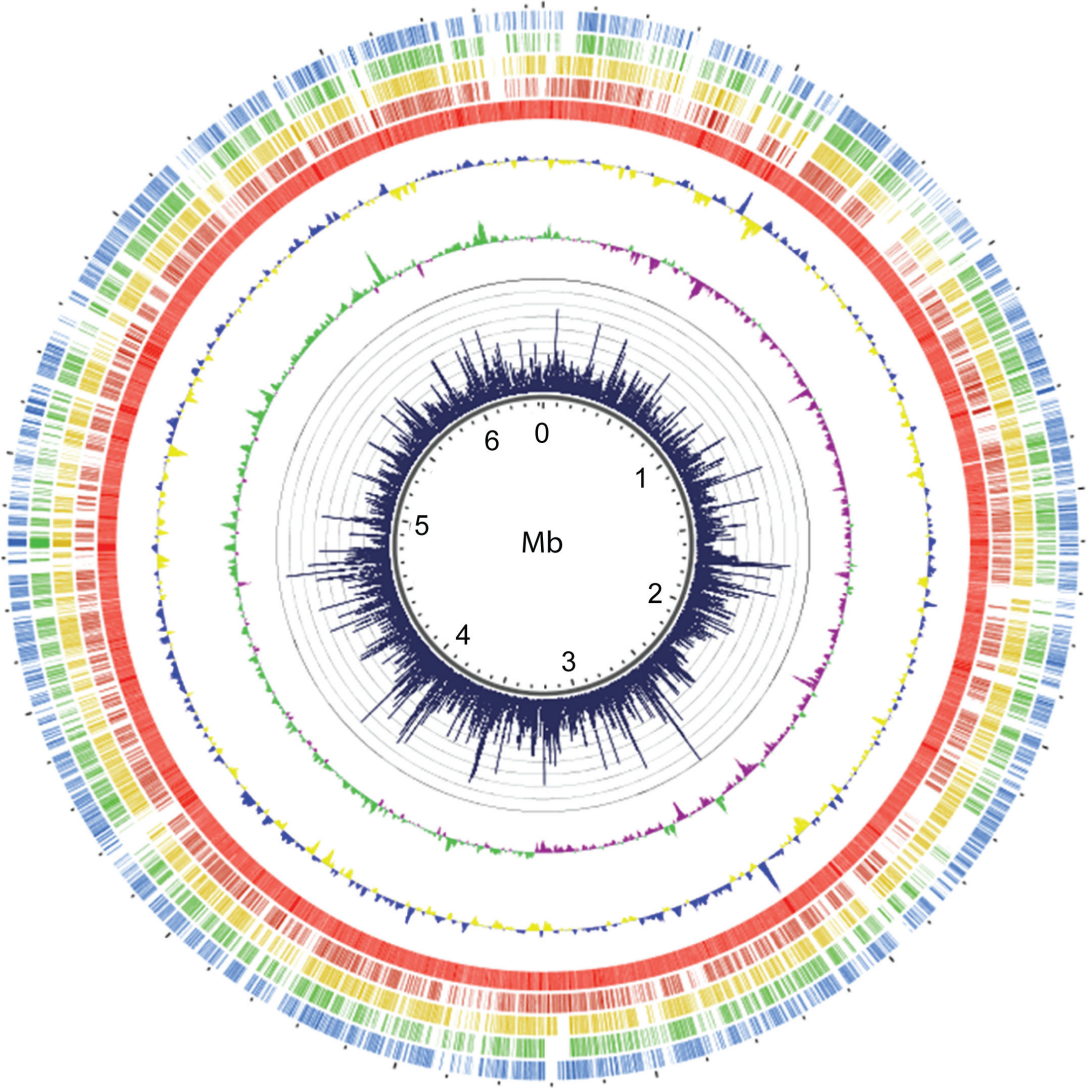
Visualization of the *M. kansasii* chromosome showing insertion counts, orthology data, and various sequence features. Tracks from innermost: (1) chromosome backbone with ruler; (2) insertion count plot with the height of the black bars representing the number of raw insertion counts at each TA site (minimum = 0, maximum = 45,000); (3) GC skew (green represents richness of G over C, purple represents richness of C over G; (4) GC content (blue represents above average GC content, yellow represents below average GC content); (5) *M. kansasii* annotated genes (bright red); (6) *M. tuberculosis* orthologs of *Mk* open reading frames (ORFs) (dark red); (7) *M. avium* subsp. *hominissuis* orthologs (yellow) of *Mk* ORFs; (8) *M. abscessus* orthologs (green) of *Mk* ORFs; and (9) *M. smegmatis* orthologs (blue) of *Mk* ORFs.

**Fig 2:**
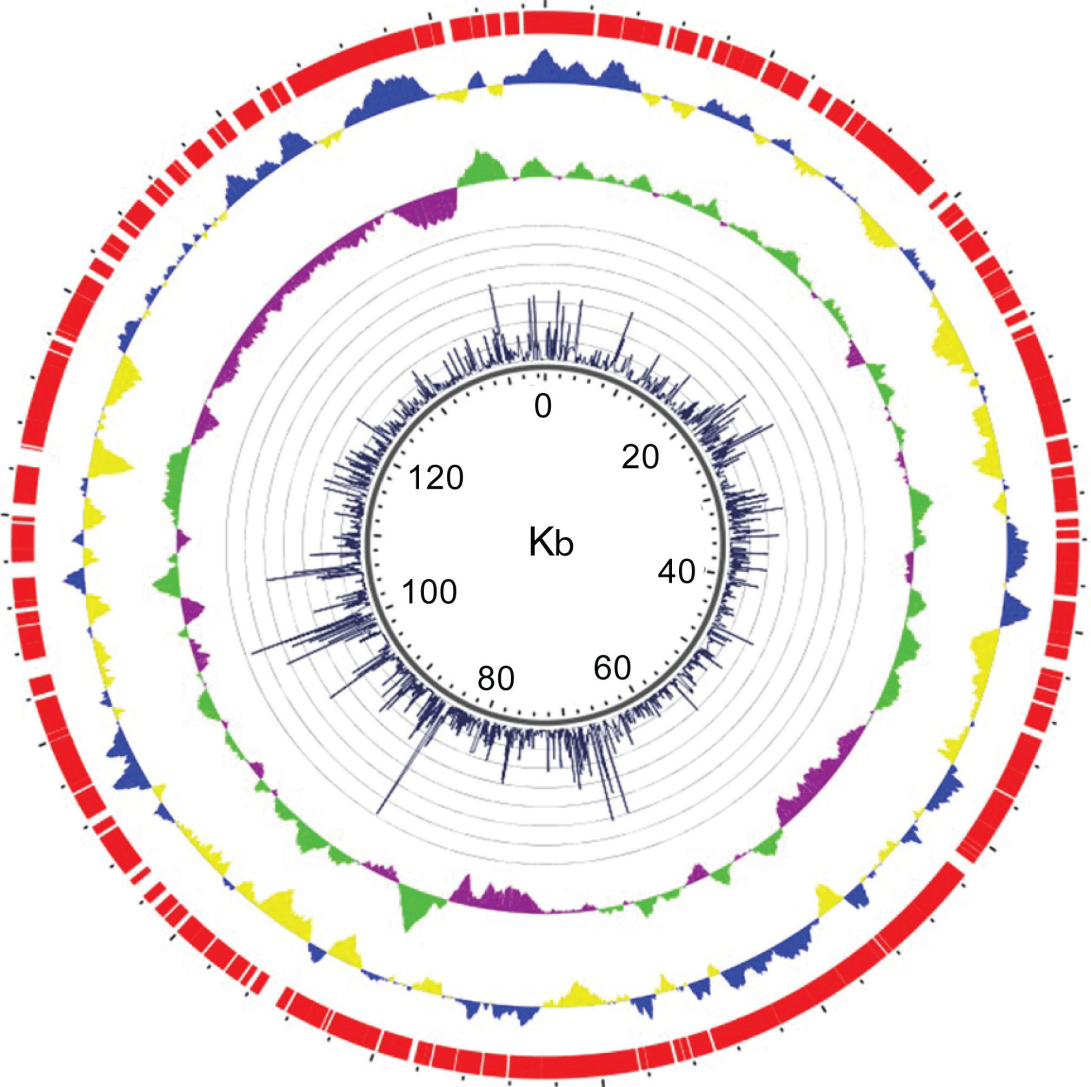
Visualization of the *M. kansasii* plasmid pMK12478 showing insertion counts and various sequence features. Tracks are as described in the legend of Fig. 1. Insertion count plot minimum = 0, maximum = 37,000.

**Fig 3:**
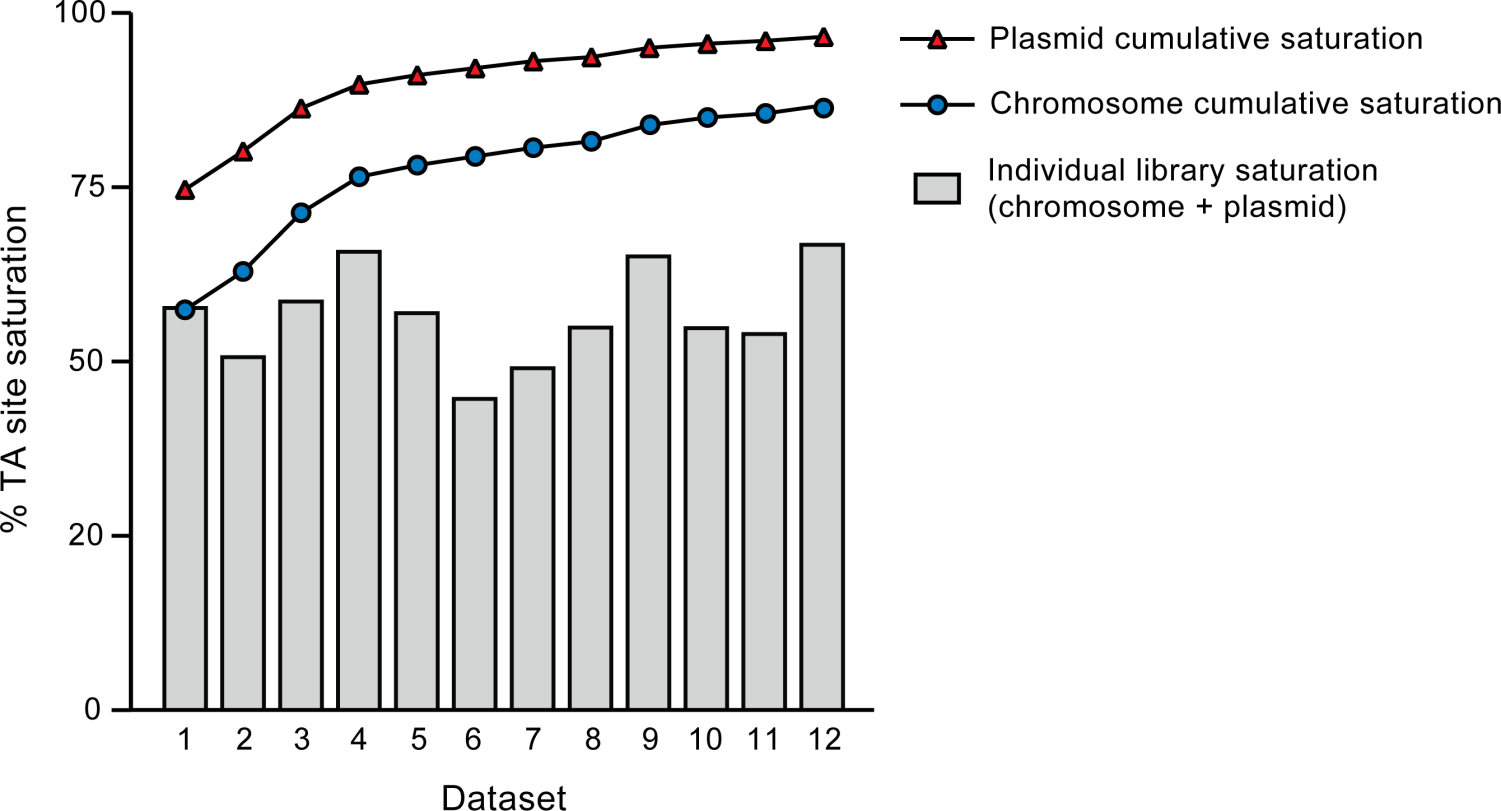
TA site insertion saturation. Each data set represents TnSeq results from a single-plated library. Gray bars show the overall saturation levels (chromosome + plasmid) of individual data sets. The solid lines represent the cumulative densities of the chromosome (circles) and plasmid (triangles) obtained by adding one data set at a time. Each data set was generated by harvesting and processing plated libraries as described in Materials and Methods. Briefly, colonies from a single library were scraped from the surface of 7H10 agar, and genomic DNA was extracted from the mutant pool. Genomic DNA was fragmented, fragment ends were repaired, A-tailed, and ligated to T-tailed adapters containing random nucleotide barcodes. PCR enrichment of fragments containing Tn-chromosome junctions using a Tn-specific primer and an adapter-specific primer was followed by the selection of fragments between 400 and 600 bp via gel electrophoresis and then a second hemi-nested PCR amplification using tailed primers to introduce Illumina-specific sequences. The resulting fragment libraries were sequenced on the Illumina platform, obtaining at least 30 million 100-bp paired-end reads per sample. Reads were processed and mapped onto the *Mk* chromosome and the *Mk* plasmid pMK12478 using the TPP tool in TRANSIT.

We performed TnSeq analysis on 12 independent libraries of *Mk* Tn mutants grown on Middlebrook 7H10 agar plates. Each library contained an average of approximately 200,000 mutant colonies. These libraries, totaling over 2.5 million colonies, yielded an average of 11 million unique Tn–genome junctions (template counts) per library (Table S1). The *Mk* genome consists of a 6,432,277-bp chromosome with 97,702 TA sites and a 144,951-bp plasmid (pMK12478) containing 2,251 TA sites. Thus, the aggregated library analyzed corresponded to a 25-fold coverage relative to the number of TA sites in the genome. Insertions were evenly distributed throughout both the chromosome and the plasmid (Fig. 1 and 2). The TA site insertion saturation (defined as the ratio of the number of TA sites with at least one mapped insertion to the total number of TA sites) for individual libraries ranged from 44.8% to 67.0% (Fig. 3). Cumulatively, 87.0% of the 99,953 TA sites in the genome (chromosome + plasmid) contained at least one insertion in the aggregated data set. This equated to 86.8% and 96.6% cumulative TA site insertion saturation for the chromosome and the plasmid, respectively. For clarity, we note that, unless otherwise indicated, further analysis addressed in the ensuing text is focused on the chromosome, rather than on the plasmid, for which a dedicated section is presented below.

The insertion data sets from 12 libraries were analyzed collectively in TRANSIT using the hidden Markov model (HMM) to assign the most probable essentiality state call to each TA site (37 - 40). This statistical model incorporates neighboring TA site read counts when assigning each TA site its essentiality state call, thus producing a locally consistent assignment of essentiality state calls across the genome. The TA essentiality state calls assigned by the HMM are essential (ES, being near zero read count), nonessential (NE, being near the mean read count), growth defect (GD, being near 1/10 of the mean), and growth advantage (GA, being near 5× the mean). In this way, the HMM is capable of identifying not only TA sites within essential regions of the genome but also those sites in regions resulting in an apparent reduction (GD) or improvement (GA) in fitness when disrupted. The use of TnSeq data to accurately predict changes in fitness associated with Tn insertions has been experimentally validated (41) and represents an advantage of the HMM component of TRANSIT over other available models for assessing the essentiality of intragenic and extragenic regions of bacterial genomes. It is worth noting that, like any other method of TnSeq analysis for predicting gene essentiality, TRANSIT has advantages and disadvantages (35, 37 - 40). Still, TRANSIT remains a well-accepted and powerful tool for gene essentiality studies with the *Himar1* Tn in the mycobacterial field.

Of the 12,899 chromosomal TA sites (13.2%) to which no insertions were mapped in our aggregate data set, 4,561 were categorized as ES by the HMM. After excluding the TA sites with ES state assignments from analysis, the overall saturation for the chromosome increases to 90.8% from 86.8% (Table 1). Additionally, our analysis identified 8.7% (8,456 TA sites) of all TA sites as fitting the nonpermissive (NP) motif for Tn insertion as defined by Dejesus et al. (35). Such TA sites are less permissible for insertion compared to TA sites not fitting the NP motif. This percentage of NP TA sites is comparable to that observed in the essentiality analysis of the *Mtb* genome, in which 9% of the TA sites fit the NP motif (35). Taking these TA sites matching the NP motif into consideration, the overall saturation increases to 94.6% from 90.8% (Table 1), leaving only 4,500 TA sites (4.6%) that were not labeled ES, did not fit the NP motif, and did not contain at least one insertion in our aggregated data set. Given the high saturation of our library, it is improbable that these many sites are not occupied in the aggregated data set purely by chance. It is more likely that these sites reside in essential regions of the genome but were not labeled ES due to the features of the HMM component of TRANSIT (see below). Overall, when applied to our TnSeq data, HMM defined 7.0% of TA sites as ES, 78.6% as NE, 2.3% as GD, and 12.1% as GA (Data set S1). Notably, this distribution of TA site essentiality state calls is very similar to that observed in essentiality analysis of the rapidly growing NTM *M. abscessus* genome (33).

**TABLE 1:** Insertion count statistics of *M. kansasii* chromosomal TA sites

TA site category	No. of sites	% of sites^*c*^	% of saturation^*d*^	NZ mean^*e*^
All	97,702	–	86.8	144.5
Predicted to not be essential by HMM^*a*^	90,873	93.0	90.8	148.5
Predicted to not be essential not matching NP^*b*^ motif	83,010	85.0	94.6	153.8
All TA sites matching NP motif	8,456	8.7	48.0	44.2
All TA sites not matching NP motif	89,246	91.3	90.5	149.6

^*a*^ Essentiality assessment using the hidden Markov model (HMM) and performed in TRANSIT (38) using TnSeq data.

^*b*^ Nonpermissive TA sites for *Himar1* insertion as defined by Dejesus et al. (35).

^*c*^ Percentage of all TA sites represented by category.

^*d*^ Percentage of TA sites in category with at least one insertion.

^*e*^ Mean read count of nonzero (NZ) TA sites in the category.

### Essentiality analysis of annotated genes

The essentiality state calls of TA sites were the basis for the essentiality analysis of the annotated genes in the *Mk* genome. Using TRANSIT, each gene was assigned the majority state call of the TA sites within its boundaries. The results of our essentiality analysis of annotated genes are presented in Table 2 and Data set S1. Genes lacking TA sites (*n* = 19) were not assessable and therefore were labeled N/A. A total of 76 genes featured a single TA site, 131 had two TA sites, and 232 contained three TA sites. While it can be challenging to assess the essentiality state of genes containing so few TA sites because of the possibility that TA sites lack insertions in the aggregate data set due to chance, only 14 genes belonging to these three groups totaling 439 genes were identified as ES by TRANSIT. Of the 5,350 annotated open reading frames (ORFs) in the *Mk* genome, 394 (7.4%) were identified as ES and 139 (2.6%) as GD (Table 2; Data set S1), representing a combined set of 533 (10.0%) ORFs that are required for optimal growth *in vitro*. As for the remaining ORFs, 649 (12.1%) were identified as GA and 4,153 (77.6%) as NE.

**TABLE 2:** Summary of essentiality analysis of *M. kansasii* chromosome by TnSeq

Genomic feature^*a*^		No. of genomic features by assigned essentiality status^*b*^				
	Total	ES	GD	GA	NE	N/A
ORFs	5,350	394	139	649	4,153	15
tRNAs	46	10 (40)^*c*^	0 (1)	5 (1)	27 (0)	4
rRNAs	3	3	0	0	0	0
ncRNA	2	1 (2)	0	0	1 (0)	0
miscRNA^*d*^	1	1	0	0	0	0

^*a*^ As per the National Center for Biotechnology Information annotation (GenBank: NC_022663.1).

^*b*^ ES, essential; GD, growth defect; GA, growth advantage; NE, nonessential; N/A, not assessable due to the lack of TA sites.

^*c*^ Numbers in parentheses are totals based on gene final essentiality state calls determined by manual scrutiny of TA site insertion data (see Data set S4 ).

^*d*^ miscRNA, miscellaneous RNA.

### Comparative landscape of gene essentiality and orthology across *M. kansasii* and other mycobacteria

**Fig 4:**
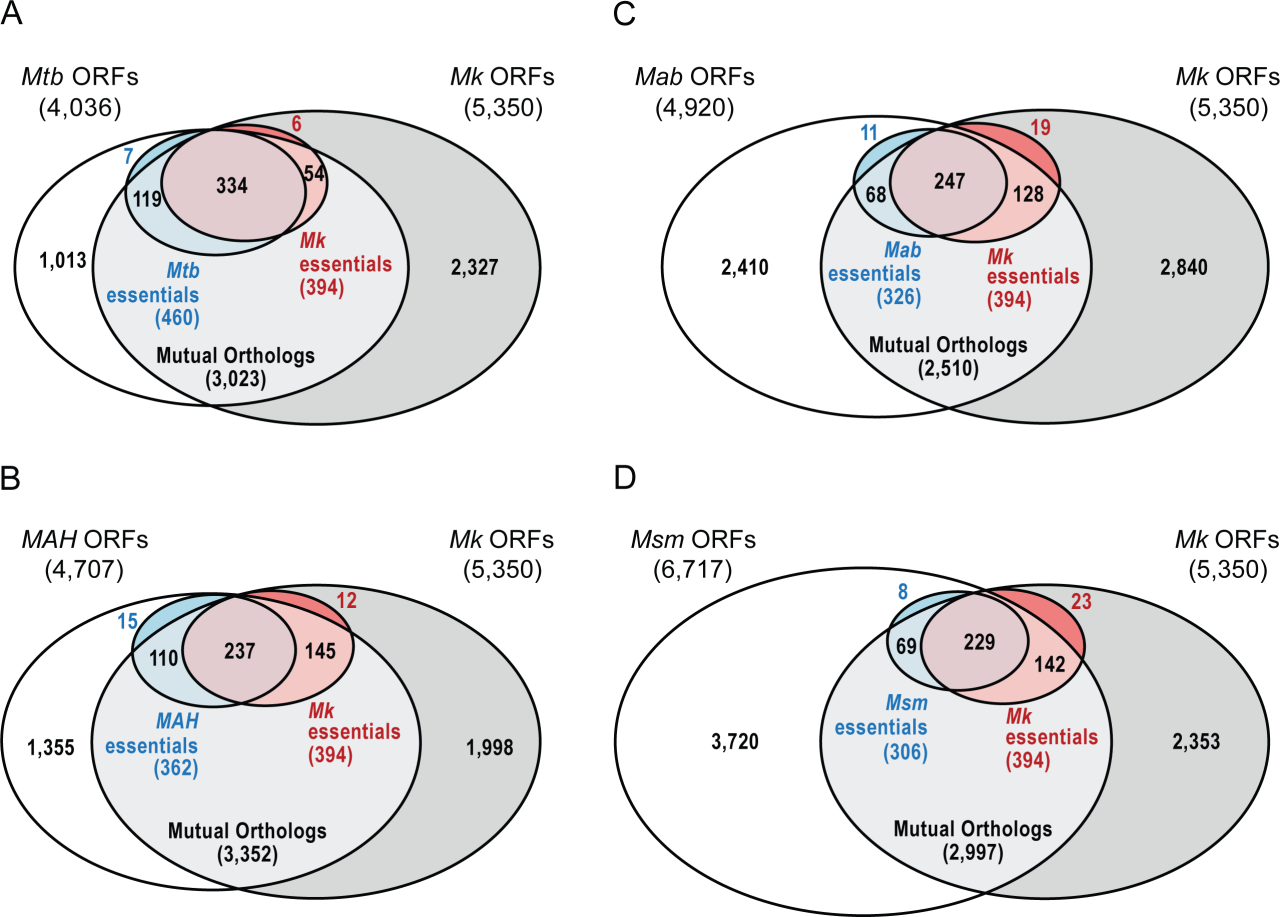
Venn diagrams illustrating *in vitro* essential ORFs of *M. kansasii* compared to other mycobacteria species. Mutual orthologs were identified using Gview server as described in Materials and Methods. (**A**) *M. tuberculosis* essential ORFs defined by Dejesus et al. (35). (**B**) *M. avium* subsp. *hominissuis* essential ORFs defined by Dragset et al. (34). (**C**) *M. abscessus* essential ORFs defined by Rifat et al. (33). (**D**) *M. smegmatis* essential ORFs defined by Dragset et al. (36). Venn diagrams were generated in R using the Eulerr package and edited in Adobe Illustrator.

We carried out pair-wise comparative genomics analysis to identify *Mk* ORFs that share mutual orthologs with either *Mtb*, *MAH*, *Mab*, or *Msm*. We note that the GView BLAST search results returned ORFs that were not present in the published essentiality data sets used for *Mtb* (18 ORFs) (35) and *MAH* (54 ORFs) (34). Further details on these ORFs and the corresponding essentiality state calls used for the purpose of this study can be found in the Materials and Methods section. Our comparative genomics analysis found that a total of 1,910 (35.7%) *Mk* ORFs shared mutual orthologs with all species compared, whereas 1,187 (22.2%) *Mk* ORFs did not share mutual orthologs with any of the four species compared. The orthology data were cross-referenced with available essentiality data for each species (Fig. 4; Table 3; Data set S2 ). A total of 139 essential *Mk* ORFs shared mutual orthologs predicted to be essential in all four species compared (Data set S3). Many of these ORFs correspond to genes involved in processes critical for survival such as DNA replication, transcription, translation, cell wall maintenance, and amino acid biosynthesis. Among this group of 139 common essential ORFs are genes encoding targets of drugs currently used for treating mycobacterial infections such as *embAB* (ethambutol’s target), *inhA* (isoniazid’s target), *rpoB* (rifampicin’s target), as well as genes encoding the targets of antitubercular compounds in various stages of development, including *dprE1* (macozinone’s target), *murX* (capuramycin’s target), *kasA* (indazole sulfonamide’s target), and *leuS* (oxaboroles’ target) (42).

**TABLE 3:** Comparison of essentiality and *M. kansasii* orthology across species

Species	Total ORFs	Essential ORFs		No. of *M. kansasii* orthologs^*e*^
		No.	% of total	
*M. kansasii*	5,350	394	7	–
*M. tuberculosis*	4,036	460^*a*^	11	3,023
*M. avium* subsp. *hominissuis*	4,707	362^*b*^	8	3,352
*M. abscessus*	4,920	326^*c*^	7	2,510
*M. smegmatis*	6,716	306^*d*^	5	2,997

^*a*^ As defined by Dejesus et al. (35).

^*b*^ As defined by Dragset et al. (34).

^*c*^ As defined by Rifat et al. (33).

^*d*^ As defined by Dragset et al. (36).

^*e*^ Orthologs identified using Gview Server as described in Materials and Methods.

Interestingly, 13 *Mk* ES ORFs shared mutual orthologs that are not essential (NE, GD, or GA) in all four species compared (Table 4). Among these ORFs uniquely essential in *Mk*, the ES status of *secA2* is of particular interest. Mycobacterial genomes encode two paralogs of SecA, the ATPase component of the major bacterial protein secretory system. In bacteria containing both SecA1 and SecA2, the former provides an essential function by exporting the majority of the bacterial secretome, whereas the latter typically becomes essential only under *in vivo* conditions and exports a smaller set of the secretome, including proteins involved in virulence (43, 44). While the available essentiality data suggest this model is generally correct for mycobacteria, our unexpected finding that the genes encoding both SecA paralogs are essential in *Mk* indicates that SecA2 might play a more critical function in *Mk* biology. SecA2 and other products of genes that are uniquely essential in *Mk* represent potential targets for *Mk*-specific drugs.

**TABLE 4:** *M. kansasii* essential ORFs sharing mutual orthologs,^*a*^ identified as not essential in *M. tuberculosis,^b^ M. avium* subsp. *hominissuis,^c^ M. abscessus,^d^* and *M. smegmatis^e^*

Locus tag	Gene name	Description
MKAN_RS00320	*secA2*	Accessory Sec system translocase SecA2
MKAN_RS14925	*–*	Cation-translocating P-type ATPase
MKAN_RS16715	*–*	Hypothetical protein
MKAN_RS17105	*thiD*	Bifunctional hydroxymethylpyrimidine/phosphomethylpyrimidine kinase
MKAN_RS18455	*rpmG*	50S ribosomal protein L33
MKAN_RS18980	*–*	zf-HC2 domain-containing protein
MKAN_RS22360	*–*	Cysteine desulfurase
MKAN_RS22365	*mnmA*	tRNA 2-thiouridine(34) synthase MnmA
MKAN_RS22965	*–*	DUF177 domain-containing protein
MKAN_RS23125	*rpsP*	30S ribosomal protein S16
MKAN_RS23140	*trmD*	tRNA (guanosine(37)-N1)-methyltransferase TrmD
MKAN_RS23630	*cobA*	Uroporphyrinogen-III C-methyltransferase
MKAN_RS24245^*f*^	*–*	DUF3093 domain-containing protein

^*a*^ Identified using Gview Server as described in Materials and Methods.

^*b*^ As defined by Dejesus et al. (35).

^*c*^ As defined by Dragset et al. (34).

^*d*^ As defined by Rifat et al. (33).

^*e*^ As defined by Dragset et al. (36).

^*f*^ Scrutiny of insertion and state call data (Data set S1) for MKAN_RS24245 revealed that three out of the six TA sites in the ORF fit the NP motif, a confounder with the potential to reduce the robustness of the ES prediction.

Seven *Mk* ORFs identified as being nonessential share mutual orthologs that are essential in all species compared (Table 5). Among these is *nadD*, encoding a nicotinate-nucleotide adenylyltransferase. The predicted essentiality of *nadD* for *in vitro* growth has been experimentally validated for *Msm*, where the *nadD* knockdown has been shown to have a bacteriostatic effect secondary to the depletion of the NAD(H) cofactor pool in the mutant (45). The unexpected prediction of *Mk nadD* as NE by our TRANSIT analysis might indicate that the *Mk* genome encodes nicotinate-nucleotide adenylyltransferase paralogs or metabolic pathway alternatives absent in the compared species that allow the bacterium to bypass the requirement of *nadD* for *in vitro* growth. It is worth noting, however, that our scrutiny of insertion and state call data (Dataset S1) for *nadD* revealed a low value for the mean normalized read count at nonzero sites (five TA sites with insertions out of seven) conceptually more consistent with a GD or ES classification of the ORF than the NE classification by TRANSIT. The NE call by TRANSIT might have been heavily influenced by the insertion pattern surrounding the ORF. Interestingly, the same observation can be made for *whiA* (*MKAN_RS25620*) (Table 5). We also note that no obvious paralogs of *nadD* or *whiA* are present in *Mk*. Experimental research will be required to ascertain the essentiality status of *Mk nadD* and *whiA* conclusively. Lastly, 1,187 *Mk* ORFs share no mutual orthologs with any species compared. Most of these (1,045) were identified as NE, while only two, encoded by *MKAN_RS27135* and *MKAN_RS30405*, were identified as ES. Both genes are predicted to encode hypothetical proteins of unknown function.

**TABLE 5:** *M. kansasii* ORFs identified as not essential sharing mutual orthologs^*a*^ identified as essential in *M. tuberculosis,^b^ M. avium* subsp. *hominissuis,^c^ M. abscessus,^d^* and *M. smegmatis^e^*

Locus tag	State call^*f*^	Gene name	Gene product description
MKAN_RS04970^*g*^	NE	*nadD*	Nicotinate-nucleotide adenylyltransferase
MKAN_RS25620^*h*^	NE	*whiA*	DNA-binding protein WhiA
MKAN_RS14030	GD	*–*	CCA tRNA nucleotidyltransferase
MKAN_RS16635	GD	*dnaK*	Molecular chaperone DnaK
MKAN_RS17640	GD	*–*	Glutamyl-tRNA reductase
MKAN_RS22580	GD	*–*	D-alanine–D-alanine ligase
MKAN_RS24890	GD	*–*	Glycosyltransferase family 4 protein

^*a*^ Orthologs identified using Gview Server as in Materials and Methods.

^*b*^ As defined by Dejesus et al. (35).

^*c*^ As defined by Dragset et al. (34).

^*d*^ As defined by Rifat et al. (33).

^*e*^ As defined by Dragset et al. (36).

^*f*^ GD, growth defect; NE, nonessential.

^*g*^ Scrutiny of insertion and state call data (Data set S1) for MKAN_RS04970 revealed a low value for the mean normalized read count at nonzero sites (five TA sites with insertions out of seven) conceptually more consistent with a GD or ES classification of the ORF than the NE prediction by TRANSIT.

^*h*^ Inspection of insertion and state call data for MKAN_RS25620 showed five unoccupied TA sites (out of six in the ORF) and a low value for the mean normalized read count at nonzero sites conceptually consistent with an ES call rather than with the NE classification by TRANSIT.

### Candidate *M. tuberculosis* essential drug targets that are not essential in *M. kansasii*

Our results indicate that *Mk* has roughly 15% fewer *in vitro* essential ORFs than *Mtb*. This finding, paired with the observation that the *Mk* genome is ~50% larger than that of *Mtb*, suggests that there are fundamental differences in physiology between the two pathogens, which, in turn, might translate into yet unrecognized differences in susceptibility to antitubercular drugs and drug candidates between the two species.

In support of this view, the gene *thyX*, which encodes one of the targets of the second-line antitubercular drug *p*-aminosalicylic acid (PAS), to which *Mtb* is susceptible but *Mk* is intrinsically resistant (17, 46, 47), was identified as NE by our analysis. PAS acts as a prodrug targeting the folate biosynthesis pathway by inhibiting the dihydrofolate reductase encoded by *folA* (*dfrA*) (48). A recent study has shown that PAS is a multitarget drug, also inhibiting the flavin-dependent thymidylate synthase encoded by *thyX* (49). While *folA* is labeled GD in both *Mtb* (*Rv2763c*) and *Mk* (*MKAN_RS23945*), *thyX* is labeled ES in *Mtb* (*Rv2754c*) (35) but NE in *Mk* (*MKAN_RS23975*). Thus, the intrinsic resistance of *Mk* to PAS is accompanied by a lack of requirement for the gene encoding one of the targets of the antitubercular agent. Future studies are warranted to probe the link between the NE status of *Mk thyX* and the resistance of the bacterium to PAS, a drug with a mechanism of action that remains incompletely understood.

One potential explanation for the reduced proportion of essential genes in *Mk* relative to *Mtb* is that the larger genome of the former encodes functionally redundant genes or pathways that are absent in the smaller genome of the latter. Functional redundancies have been observed in *Mtb* (50 - 53) and have occasionally confounded the validation of antitubercular agents (54). Therefore, it is possible that any additional layers of redundancy in the *Mk* genome could confound the prediction of drug targets in *Mk* made by the extrapolation of *Mtb* knowledge and hinder the activity of antitubercular agents validated in *Mtb* against *Mk*. To probe this possibility, we mined the results of our comparative genomics analysis and essentiality study to predict potential differences in drug target candidates between *Mtb* and *Mk*. In the ensuing text, we highlight candidate *Mtb* essential drug targets sharing mutual orthologs with *Mk* that we identified as nonessential. These findings indicate the presence of additional pathways or proteins that negate these drug target candidates in *Mk* and may have implications for the decision-making process shaping the drug candidate evaluation pipeline against this pathogen.

Among the noticeable discrepancies in gene essentiality between *Mk* and *Mtb* with possible implications for drug development are the essentiality assertions for genes encoding proteins of the proteasome machinery, a critical component of the *Mtb* proteostasis network (55, 56). Gene-silencing experiments indicate that while the *Mtb* proteasome is not essential under standard liquid-culturing conditions *in vitro*, it is required for optimal growth on agar plates and for growth and persistence in mice (57, 58). These findings have made the *Mtb* proteasome an attractive target for the development of anti-TB drugs (59 - 61). In *Mtb*, genes *prcA* (*Rv2109c*) and *prcB* (*Rv2110c*) encoding the proteasome subunits PrcA and PrcB, respectively, as well as *Rv3780* encoding the bacterial proteasome activator/accessory factor PafE, were identified as ES (35). Our essentiality analysis, which was carried out using the same solid medium as that of the *Mtb* study, identified *MKAN_RS02165* and *MKAN_RS02170*, encoding *Mk* PrcA and PrcB, respectively, as NE, while the *MKAN_RS13390*, encoding *Mk* PafE, was identified as GA. Notably, however, none of the three *Mk* genes appear to have identifiable paralogs in the genome. Thus, our findings might suggest that *Mk* possesses at least one alternative pathway or compensatory mechanism not present in *Mtb* that is sufficient to fulfill the essential protein degradation needs experienced by the cell during growth in solid medium in the absence of a conventional mycobacterial proteasome assembly. Whether or not this pathway(s)/mechanism(s) is sufficient to render these *Mk* proteasome genes dispensable during the conditions encountered in a mammalian host remains to be determined.

Another promising anti-TB drug target candidate is the serine/threonine protein kinase A (PknA), which phosphorylates proteins involved in many critical mycobacterial cell processes such as mycolic acid synthesis, peptidoglycan synthesis, and cell division (62). The *Mtb* gene *pknA* (*Rv0015c*) has been shown to be indispensable for survival in the host using a conditional knockout mutant in a mouse model (63) and identified as ES for *in vitro* growth by TnSeq-based essentiality analysis (35). Of note, a recent large-scale screen of Food and Drug Administration–approved drugs for *Mtb* PknA inhibitors identified existing vitamin B2-based drugs as candidates to feed the pipeline of preclinical studies on the PknA target (64). In contrast to the finding of the TnSeq-based essentiality analysis in *Mtb*, our data revealed that *Mk pknA* (*MKAN_RS14245*) disruption provides a growth advantage rather than a loss of viability (Data set S1). Although the *Mtb* and *Mk* genomes encode several serine/threonine protein kinases, *Mk* does not appear to have any additional PknA paralog that could explain the difference in essentiality between the two pathogens. Besides *pknA*, the only other serine/threonine protein kinase identified as essential in *Mtb* is *pknB* (35). Interestingly, we found that *Mk pknB* (*MKAN_RS1440*) was not essential for growth, but its disruption led to a growth defect (Data set S1). It is worth noting that in *Mtb*, PknA and PknB regulate the activity of the proteasome by phosphorylating the core subunits PrcA and PrcB (65), suggesting that the disagreements in the gene essentiality call for *pknA*, *pknB*, and proteasome subunit encoding genes *prcA* and *prcB* of *Mtb* and *Mk* might be linked to fundamental differences in the way the two pathogens manage their respective proteostasis networks.

Our analysis also suggests differences in potential target candidates for drugs disrupting energy metabolism. Type II NADH-dehydrogenase (NDH-2) plays a critical role in bacterial energy metabolism as it catalyzes the transfer of electrons from NADH to the respiratory chain (66, 67). The *Mtb* genome contains two NDH-2 genes, *ndh* (*Rv1854c*) and *ndhA* (*Rv0392c*), encoding NDH-2 and NDH-2A, respectively. Early gene knockout studies in *Mtb* found that *ndh* could not be inactivated without an additional copy of the gene present (68), while a more recent study showed that successful deletion of *ndh* results in a growth defect *in vitro* (69). In contrast, *ndhA* could be inactivated without compromising growth (68, 69). The absence of an NDH-2 ortholog in mammals has made it an attractive target for new antibiotics, and several chemical library screens have identified inhibitors of the mycobacterial NDH-2 (42, 70 - 73). Essentiality analysis in *Mtb* identified *ndh* as GD and *ndhA* as NE (35), supporting the findings from the gene knockout studies. Our analysis identified *MKAN_RS00490* (encoding the *Mk* ortholog of NDH-2) and *MKAN_RS16920* (encoding the *Mk* ortholog of NDH-2A) as NE and GA, respectively. This may represent a potential difference in energy metabolism between *Mk* and *Mtb*. Both species contain the nonessential proton-pumping type I NADH-dehydrogenase (NDH-1) encoded by *nuoA-G*. While data suggest that NDH-1 is unable to complement the loss of NDH-2 function in *Mtb* (70), it is possible that NDH-1 and/or NDH-2A can better compensate the loss of NDH-2 in *Mk*.

Nonessential *MKAN_RS23135* encodes ribosome maturation factor RimM, which shares mutual orthologs identified as GD in all four species compared. Data obtained from analysis of RimM knockout mutants (74) and mutational analyses of ribosomal components in *E. coli* (75), along with kinetic experiments (76), indicate that RimM plays an important role in ribosome biosynthesis in bacteria. The observation that a *rimM* gene knockout significantly impacts bacterial growth (77) and that humans lack an ortholog of RimM has led to recent studies of the protein as a potential drug target in *Mtb*. These studies have provided the structural basis for *Mtb* RimM–ribosomal protein S19 interaction and predicted the druggability of the resulting complex (78). Again, we found no apparent paralogs of RimM in the *Mk* genome to explain the lack of requirement of its gene for wild-type growth *in vitro*. Thus, our analysis suggests that RimM may not be a viable drug target for combatting infections by the opportunistic pathogen.

Many species of bacteria, including *Mtb*, utilize the methylerythritol phosphate (MEP) pathway to synthesize isopentenyl pyrophosphate and dimethylallyl pyrophosphate, which are the precursors to isoprenoids (79). The roles of isoprenoids in a wide variety of critical mycobacterial cellular functions and a lack of the MEP pathway in humans have led to investigations into the druggability of the pathway in *Mtb*. The genes encoding the seven enzymes in the MEP pathway (*Rv2682c*/1-deoxy-D-xylulose 5-phosphate synthase [DXS], *Rv2870c*/IspC, *Rv3582c*/IspD, *Rv1011*/IspE, *Rv3581c*/IspF, *Rv2868*/IspG, *Rv1110*/IspH) were identified as ES in *Mtb* (35), and thus each represents a potential drug target. However, the biochemical properties of some of these enzymes have complicated the design of inhibitors, and as a result, DXS, IspC, and IspF have emerged as the most promising targets in the MEP pathway (80). In *Mk*, the mutual orthologs of DXS and IspC, MKAN_RS24340 and MKAN_RS23400, respectively, were identified as ES, while the mutual ortholog of IspF, MKAN_RS11675, was identified as GA. Therefore, among the inhibitors of potential drug targets in the *Mtb* MEP pathway, those designed to target IspF may not be active against *Mk*. It should be noted that in the case of *ispF* and the gene *rimM* (discussed above), most insertions mapped to TA sites toward the 3′-end of the ORFs. It remains possible that the insertions compromised only the C-terminus of the gene products and render functional proteins. Thus, the essentiality call by TRANSIT might be confounded and it is possible that these genes are indeed essential. Unambiguous experimental validation of the essentiality status of these genes is needed.

The *Mtb* essential gene *desA1* (*Rv0824c*) encodes an acyl-acyl carrier protein desaturase that has been identified as a high-confidence drug target by an *in silico* target identification pipeline (81). Studies using the model organism *M. smegmatis* have elucidated the role of DesA1 in the desaturation step of mycolic acid biosynthesis and have highlighted its potential as a new antitubercular target (82, 83). *Mtb* DesA1 shares a mutual ortholog in *Mk*, encoded by *MKAN_RS10190* (henceforth referred to as *Mk* DesA1), which was identified as GA. The presence of an apparent paralog of *Mk* DesA1 in the *Mk* genome encoded by the NE gene *MKAN_RS04740* (sharing 77.8% identity with *Mk* DesA1) may explain this lack of requirement of DesA1 in *Mk*. This may have implications for the validation of drugs targeting DesA1 in *Mk*.

### Essentiality of non-protein-coding genes

The *Mk* genome contains 46 tRNA genes, of which 10 were predicted as ES by TRANSIT (Table 2; Data set S4). Somewhat unexpectedly, these essential genes included only two of the eight singleton tRNA genes, tRNA-Asp (*MKAN_RS10135*) and tRNA-Phe (*MKAN_RS10140*). Additionally, there are several cases where only one of the multiple copies of the tRNA genes for a given amino acid was classified as ES (i.e., Ala, Glu, Gly, Ile, Lys, Met, Thr, and Val tRNAs). This pattern of essentiality for tRNA genes is similar to what was reported for *Mab* (33) but differs from what was observed in *Mtb*, in which 21 of the 35 tRNA genes with at least two TA sites were classified as ES (35). Notably, manual scrutiny of our TA site insertion and essentiality call data for the *Mk* tRNA genes along with the equivalent data in the *Mtb* study revealed a similar pattern in both data sets. There were many instances where the TA sites within the boundary of tRNA genes are devoid of insertions (or have marginal read counts), but these sites were labeled NE by HMM. Given that the gene essentiality state given by the software is called based on the labeling of the majority of TA sites within the gene, we would expect such tRNA genes to be labeled NE in both species. The fact that many of these *Mtb* tRNA genes were reported as being ES leads us to believe that the ES classification might have emerged from a sensible manual curation performed by the authors. This “miscalling” of TA sites by HMM stems from an occasionally disadvantageous feature of the software tool, where short stretches of TA sites are forced into a state call based on the information from surrounding TA sites. Since tRNA genes tend to be relatively short and have few TA sites, their essentiality state calls are particularly susceptible to the state call information from the surrounding TA sites. Taking this all into consideration, the essentiality landscape of *Mk* tRNA genes appears to be similar to that of *Mtb* in that the majority of these genes should be considered ES. This essential status would not be surprising given the role of tRNAs in translation and other critical cellular processes (84). It is worth considering that the miscalling of the essentiality status of TA sites might have confounded some of the tRNA gene essentiality classifications in published work using the TRANSIT tool. In fact, this appears to be the case in the classification of tRNA genes reported in the *Mab* essentiality study (33) as per our examination of the published TA site insertion and essentiality call data sets.

The three ribosomal RNA genes, *MKAN_RS06025* (5S rRNA gene), *MKAN_RS06030* (23S rRNA gene), and *MKAN_RS06035* (16S rRNA gene), as well as *MKAN_RS29940* (*ssrA*), predicted to encode the transfer-messenger RNA (tmRNA) of the *trans*-translation system, and *MKAN_RS29660* (*ffs*), predicted to encode the small-RNA component of the signal recognition particle ribonucleoprotein complex, were identified as ES. It should be noted that *ffs* contains only a single TA site, and thus there is a chance that the gene is not truly essential. Interestingly, unlike *Mk ssrA*, *Mtb ssrA* has been classified as NE by TnSeq-based analysis (35), a finding in conflict with the essential status assigned to the *Mtb* gene based on the observation that it could not be deleted without the presence of a second functional *ssrA* copy integrated elsewhere in the genome (85). Our closer analysis of the *Mtb* TA site essentiality data from which the NE status of *Mtb ssrA* was determined revealed that the analysis was performed with a now outdated annotation of *ssrA* that incorrectly made the gene 239 bp longer compared to the current annotation. The erroneous annotation placed five TA sites permissive to insertion within the boundaries of the gene. The currently annotated shorter *Mtb ssrA*, however, is the same length as its *Mk* ortholog, and all the TA sites within the gene boundaries are defined as ES as per the *Mtb* TA site essentiality data (35). Therefore, we conclude that *Mtb ssrA* should be listed as ES, a revision of status that clears up the discrepancy between the reported *Mtb* essentiality analysis and the observation of the *Mtb ssrA* deletion studies.

### Essentiality of intergenic regions

18,673 TA sites (~19%) of those in the chromosome are in regions outside of annotated genes. We scanned these intergenic regions for segments containing runs of four or more consecutive unoccupied TA sites (referred hereafter to as CUTA segments); that is, no insertions are mapped to these sites in the aggregated data set. This criterion to define CUTA segments is comparable to the operational criterion to define essential regions in the *Mtb* essentiality analysis (35). CUTA segments were identified in 57 intergenic regions of the *Mk* chromosome, and only 13 of them included TA sites fitting the NP motif (Data set S5). Surprisingly, only six of the CUTA segments were adjacent to the 5′-end of essential genes, while the majority were adjacent to the 5′-end of nonessential genes. Further, 21 of the CUTA segments were between divergently oriented nonessential genes. While the presence of CUTA segments adjacent to the 5′-end of essential genes could be due to the Tn insertion-derived disruption of gene transcription, the CUTA segments adjacent to the 5′-end of nonessential genes require a different explanation. One possible mechanism leading to these CUTA segments could be the coating of the DNA by DNA-binding proteins that block transposition into the TA sites, a phenomenon decoupling the lack of insertions at those TA sites from site essentiality status. This speculation is supported by the finding that nucleoid-associated proteins (NAPs, a.k.a. bacterial chromatin proteins or histone-like proteins) that preferentially coat AT-rich sequences have been shown to hinder Tn insertion at their binding sites (86, 87). On the other hand, a second mechanism could be envisioned in which insertion at these TA sites is unobstructed and leads to loss of cell viability due to the disruption of the binding of NAPs or other proteins needed for suppressing deleterious (over)expression or misexpression of the adjacent nonessential gene. In this mechanism, unlike the first one noted, the lack of insertion will remain linked to the TA site essentiality status of the intergenic region.

NAPs are known to influence the expression of hundreds of genes in bacterial genomes, often by impacting the architecture of the bacterial chromatin (88, 89). In particular, several NAPs have been identified in mycobacteria that share orthologs in *Mk* (EspR, Lsr2, HupB, mIHF, and NapA/*M*) as per our comparative genomics analysis. Some of these NAPs preferentially bind AT-rich sequences, such as those found in promoter regions, and repress gene expression through DNA coating and/or introduction of local structural changes in the DNA (90 - 92). For example, the mycobacterial NAP Lsr2 preferentially binds regions with an AT content of ~53% or more, a percentage well above the 33%–35% average for mycobacterial genomes. In fact, the intergenic regions containing CUTA segments have an average AT content of 43%, which is higher than that across the entire *Mk* chromosome (24). It has been observed that in bacterial genomes, intergenic regions generally contain higher AT content than intragenic regions, but this difference is less marked in mycobacteria (93, 94).

Interestingly, 14 of the CUTA segments identified were located adjacent to the 5′-end of genes encoding members of the PE/PPE protein families (Data set S5), a group of mycobacterial secreted proteins involved in a wide range of processes, including iron uptake, adaptation to stress, and virulence (95 - 97). The *Mk* chromosome is predicted to encode 72 PE and 141 PPE proteins (24). Interestingly, while these 213 genes account for only ~4% of the genes of the genome, they were found downstream of about 25% of the CUTA segments (Data set S5). The NAP Lsr2 has been shown to regulate the expression of a large number of *pe*/*ppe* genes in *Mtb* (90, 92, 98). Two of these genes are the cotranscribed *Rv3622c* and *Rv3621c* encoding PE32/PPE65, which have been shown to be involved in modulating the antimycobacterial host immune response (99). *Mtb* PE32/PPE65 share mutual orthologs in *Mk* encoded by *MKAN_RS11920/MKAN_RS11915*, which are adjacent to an identified CUTA segment. This finding provides conceptual support for the possibility that, as speculated above, *Mk* NAPs might bind to some of the CUTA segments identified by our analysis. Lastly, currently unrecognized non-coding RNA (ncRNA)/small RNA (sRNA) coding genes or regulatory elements such as riboswitches may also explain the presence of CUTA segments. Research to investigate the molecular underpinnings of these segments will expand our insight into the biology of *Mk*.

### Essentiality analysis of the plasmid

*Mk* ATCC 12478 contains pMK12478 (GenBank: NC_022654.1), a pRAW-like plasmid similar to those present in other slow-growing mycobacteria (100). Our analysis mapped insertions to 96.6% of the 2,251 TA sites on the plasmid. Of these sites, HMM classified 0.7% (15) as ES, 86.0% (1,935) as NE, 2.5% (57) as GD, and 10.8% (244) as GA (Data set S6). When TA sites labeled ES (15) and those fitting the NP motif (143) are taken into consideration, the insertion saturation increases to 98.0%. This leaves only 41 TA sites (2.0%) that were not labeled ES, did not fit the NP motif, and did not contain at least one insertion in our aggregated data set.

Of the 145 annotated ORFs for pMK12478, one was identified as ES, two as GD, 121 as NE, and 21 as GA by TRANSIT (Data set S6). The single essential gene on the plasmid, *MKAN_RS28235*, contained all of the 15 TA sites that were defined as ES (Data set S6). The essential call for *MKAN_RS28235* could signify that disruption of the gene compromised plasmid maintenance, cell growth, or both. However, the observation that some *Mk* (subtype I) clinical isolates do not contain the plasmid suggests that *MKAN_RS28235* is not required for growth (24). Moreover, our BLAST analysis indicated that the gene might encode a protein with nucleotidyltransferase activity, a property that has been documented for several plasmid replication proteins (101, 102). Thus, the lack of the plasmid in several *Mk* strains and the results of the BLAST analysis suggest that *MKAN_RS28235* is likely to be required for plasmid maintenance.

We performed a comparative analysis to identify mutual orthologs between pMK12478 and the pRAW-like plasmid pMD2 from *MAH* strain 11 (78 kb, 66 ORFs, GenBank: CM009839.1) (Data set S7), for which TnSeq and TRANSIT-derived essentiality data became available recently (34). Of the 49 mutual orthologs identified by our analysis, 47 were assigned the same essentiality state call in the two species (Data set S7). The orthology analysis did not identify an ortholog of the ES *MKAN_RS28235* in pMD2. However, BLAST analysis revealed that proteins identical to that encoded by *MKAN_RS28235* are present in several other mycobacterial plasmids, including pMAC109a of *MAH* strain 109 (GenBank: NZ_CP029333.1). A recent *MAH* 109 essentiality analysis using a rank-based filter procedure (103) identified the *MKAN_RS28235* ortholog, *DFS55_RS24595* (formerly *DFS55_24600*), as ES. As in the case of *MKAN_RS28235*, it is likely that *DFS55_RS24595* is required for plasmid maintenance.

The two GD genes identified on pMK12478, *MKAN_RS28290* and *MKAN_RS29045*, are both predicted to encode hypothetical proteins and share mutual orthologs on pMD2. The ortholog of *MKAN_RS28290* on pMD2 is the GD gene *B6K05_25320* (Data set S7). BLAST analysis shows that *MKAN_RS28290* also has an ortholog on pMAC109a, *DFS55_RS24640* (formerly *DFS55_24645*), that was identified as ES (103). The proteins encoded by *MKAN_RS28290*, *B6K05_25320*, and *DFS55_RS24640* each share 53% amino acid sequence identity with the putative replication protein RepA encoded by the gene *MAH_p01* found in the pRAW-like plasmid pMAH135 of *MAH* strain TH135 (GenBank: AP012556.1 [104]), for which no essentiality data are available. The identification of *MKAN_RS28290* and its orthologs as GD or ES genes likely indicates that their disruption compromises plasmid maintenance.

The other GD gene, *MKAN_RS29045*, shares a NE mutual ortholog on pMD2, *B6K05_25280*. BLAST analysis revealed that MKAN_RS29045 shares 64% amino acid sequence identity with the protein L842_6123 of *Mycobacterium intracellulare* (strain MIN_052511_1280, GenBank: JAON01000021.1), which is annotated as encoding a type III secretion system outer membrane O domain protein. MKAN_RS29045 shares an identical ortholog on pMAC109a, encoded by *DFS55_RS25835*. There are no essentiality data available for this gene, which was not yet annotated in the record used for the corresponding essentiality analysis (103). However, our examination of the corresponding TA site essentiality data showed suppressed insertion counts at the gene locus, a finding suggesting the classification of *DFS55_RS25835* as a GD gene.

Interestingly, a relatively large intergenic region (828 bp) between the divergent NE *MKAN_RS29040* and *MKAN_RS28325* genes showed a run of 18 consecutive TA sites defined as GD. *MKAN_RS28325* is directly upstream of the GD *MKAN_RS29045* gene, and it is predicted to encode a member of the ParA protein family, a group of proteins known to be required for proper plasmid segregation during cell division (105, 106). The *MKAN_RS28325* ortholog on pMD2, *B6K05_25285*, was also identified as NE, whereas the ortholog on pMAC109a, *DFS55_RS24675* (formerly *DFS55_24680*), was identified as ES. Our analysis of the essentiality data for the pMAC109a region corresponding to the GD intergenic region identified upstream of *MKAN_RS28325* also showed suppressed TA site insertion counts (103). Interestingly, our RNA sequencing data from *Mk* total RNA (not shown) indicate that there is transcription at this intergenic region. Still, we were not able to identify a likely gene product by BLAST analysis of the region. Nevertheless, our findings suggest that the intergenic region is important for plasmid maintenance.

Nine of the 21 pMK12478 GA genes share mutual orthologs with pMD2 that were also identified as GA. Interestingly, these nine genes belong to two distinct loci. One locus has a cluster of five consecutive genes (*MKAN_RS28130-MKAN_RS 28150*) that are part of a putative mycobacterial type VII protein secretion (ESX) system. Such ESX loci present on pRAW-like plasmids have been shown to be required for plasmid conjugation (100). The other four GA genes are also arrayed consecutively (*MKAN_RS28295-MKAN_RS 28310*). One of these genes encodes a PPE domain-containing protein (*MKAN_RS28300*), a second encodes a putative ESX system secretion target (*MKAN_RS28305*), and the other two genes (*MKAN_RS28295* and *MKAN_RS28310*) encode hypothetical proteins. It is worth noting that the genes clustered in the two GA loci of pMK12478 appear to be conserved in pRAW-like plasmids (100), and the ESX locus is also present on pMAC109a. It remains unclear whether the GA state call for pMK12478 genes signifies that the disruption of the genes leads to an increase in the number of plasmids per cell, a growth advantage, or both.

### Conclusions

Despite the clinical relevance of *Mk* as one of the most pathogenic NTM and the need for new and more effective drugs to combat *Mk* infections, very few studies have probed the genetics of this slow-growing opportunistic pathogen. Here, we report the first comprehensive essentiality analysis of the *Mk* genome via saturation Tn mutagenesis. Our analysis, using 2.5 million *Mk* Tn mutant colonies across 12 independent plated libraries, reached an effective saturation of 94.6% of the 97,702 TA sites in the *Mk* chromosome. This level of saturation allowed us to obtain high-confidence essentiality state calls of the TA sites and annotated genes of the *Mk* genome and enabled us to compare our data to essentiality data available for other species of mycobacteria generated using similar methods.

Our comparative genomic analysis identified 139 genes that are essential in *Mk*, *Mtb*, *MAH*, *Mab*, and *Msm*. These included genes encoding targets of validated antitubercular drugs as well as targets of candidate drugs in various stages of preclinical development. The analysis also predicted unique *in vitro* genetic requirements that distinguish *Mk* from the other species of mycobacteria. Noteworthy were genes uniquely essential to *Mk*, such as *secA2*, encoding an accessory secretory system translocase, as the products of these genes may represent potential *Mk*-specific drug targets.

Importantly, our analysis highlighted candidate *Mtb* essential drug targets that are not essential in *Mk*. The lack of genetic requirement of the genes encoding these targets may have implications for the validation of drug candidates in the antitubercular drug development pipeline against *Mk* and underscores physiological differences between *Mk* and *Mtb*. Additional physiological differences between *Mk* and *Mtb* are seen by comparing the genetic requirement of the genes of the ESX loci across species (Data set S8). The *Mk* genome contains all five ESX loci present in the *Mtb* genome. For the most part, the genetic requirement of the loci is the same in *Mk* and *Mtb*. The ESX-3 locus is involved in iron and zinc homeostasis (107) and is the only locus identified as ES for *in vitro* growth in both organisms. In contrast, the ESX-5 membrane component genes *eccB5*, *eccC5*, *eccD5*, and *eccE5* were all identified as GD in *Mtb* but were classified as ES in *Mk*. The ESX-5 locus has been shown to modulate the macrophage response in slow-growing *M. marinum* (108) and appears to be the only cluster that is present in all slow-growing mycobacteria yet absent in all rapidly growing mycobacteria analyzed so far (109). An *Mtb* ESX-5 mutant in which *eccD5* was inactivated has been generated and was shown to display a wide range of phenotypes, including impaired secretion of ESX-5 substrates, reduced cell wall integrity, increased sensitivity to hydrophilic antibiotics, and attenuated growth in two infection models (110). Our essentiality data suggest the phenotypic effects resulting from disruption of one of the ESX-5 membrane components are more severe in *Mk* than in *Mtb* under *in vitro* conditions.

Our extended examination of essentiality data for TA sites in intergenic regions revealed several chromosomal regions that could contain so-far unidentified essential features or be heavily coated by DNA-binding proteins blocking transposition at these sites. Finally, the presented analysis also provided robust gene essentiality data for the *Mk* plasmid pMK12478 and its comparison with essentiality data available for other mycobacterial plasmids. In particular, the analysis highlighted several genes and an intergenic region potentially involved in plasmid maintenance.

Although data sets of *in vitro* and *in vivo* gene essentiality are likely to overlap only partially and there are well-known challenges in translating *in vitro* gene essentiality data into drug development programs, *in vitro* essentiality remains a well-recognized starting point in the process of potential drug target candidate identification and prioritization. Altogether, the genome-wide essentiality analysis of *Mk* and the results of the cross-species essentiality analysis presented herein provide novel insights into *Mk* biology and represent valuable resources to guide future *Mk* studies and to assist the process of identifying and prioritizing potential *Mk* drug target candidates.

## MATERIALS AND METHODS

### Culturing conditions and reagents

Unless otherwise specified, *Mk* (reference strain ATCC 12478; Hauduroy) and *Msm* (strain MC^2^155) were grown in Middlebrook 7H9 liquid broth (Difco, Becton-Dickinson and Co., Franklin Lakes, NJ, USA) supplemented with 10% ADN (5% BSA, 2% dextrose, 0.85% NaCl), 0.05% Tween-80, and 0.2% glycerol (s7H9) and in Middlebrook 7H10 or 7H11 agar media (Difco, Becton-Dickinson and Co.) supplemented with 10% OADC (0.05% oleic acid, 5% BSA, 2% dextrose, 0.003% catalase, 0.85% NaCl) and 0.5% glycerol (s7H10 and s7H11 media). Kanamycin (Km) was added when required to a final concentration of 30 µg/mL. Unless otherwise stated, reagents were purchased from Thermo Fisher Scientific Inc. (Branchburg, NJ, USA), New England Biolabs Inc. (Ipswich, MA, USA), VWR International LLC (Radnor, PA, USA), Sigma-Aldrich Inc. (St. Louis, MO, USA), Thomas Scientific (Swedesboro, NJ, USA), or Qiagen LLC (Germantown, MD, USA).

### Construction of *M. kansasii* transposon mutant plated libraries

High-density *Mk* Tn mutant pools were prepared as described by Budell et al. (26). *Msm* strain MC^2^155 was used as a host to propagate the φMycoMarT7 bacteriophage. s7H9 broth was used to culture host cells at 37°C with shaking for 24 hours. The resulting phage stocks were titrated on s7H11 agar plates incubated at 37°C for 48–72 hours. Titrated phage stocks were used to infect *Mk* cultures at desired PFU-to-CFU multiplicity of infection ratios. After 4 hours of incubation at 37°C without shaking, infections were pelleted, resuspended in s7H9 broth containing 25% glycerol, and aliquoted for storage at −80°C. For each infection, a single aliquot was thawed and used for titrating CFU on s7H11 agar ± Km to determine total and Km-resistant CFU. Plated libraries for the purpose of performing TnSeq were prepared using titrated *Mk*-phage infections. For each library, infections were plated to obtain ~200,000 Km-resistant CFU across 25–30 150-mm Petri dishes containing s7H10 agar with Km. Plates were incubated at 37°C for 14–18 days before colony harvesting.

### Tn insertion sequencing and essentiality analysis

Each library consisting of ~200,000 *Mk* Tn mutant colonies was processed as described by Long et al. (40). Briefly, colonies from a single library were scraped from the surface of s7H10 agar into a 50-mL conical tube. A homogeneous slurry was prepared from the cell mass by resuspension up to 30 mL in TE (Tris-EDTA) buffer. Genomic DNA (gDNA) was isolated from this mutant pool according to a standard mycobacterial gDNA isolation protocol (111). For each library, 10 µg gDNA was fragmented via ultrasonication using the Covaris E220 (Weill Cornell Genomics Core). DNA fragment ends were repaired using the End-It DNA-Repair Kit (Lucigen, LGC Biosearch Technologies Inc., Dexter, MI, USA), A-tailed with Taq-DNA polymerase (Denville Scientific Inc., South Plainfield, NJ, USA), and then ligated with T4 DNA ligase (New England Biolabs Inc.) to T-tailed adapters containing random nucleotide barcodes (Adapters 1.2 and 2.2 BarB). PCR enrichment of fragments containing Tn-chromosome junctions using a Tn-specific primer (T7) (40) and an adapter-specific primer (JEL_AP1) (40) was followed by the selection of fragments between 400 and 600 bp via gel electrophoresis and then a second hemi-nested PCR amplification using tailed primers (SOL_AP1_tagged) (40) to introduce Illumina-specific sequences. The resulting fragment libraries were sequenced on the Illumina platform (Weill Cornell Genomics Core), obtaining at least 30 million 150-bp paired-end reads per sample. Reads were processed and mapped onto the *Mk* chromosome (GenBank: NC_022663.1) and plasmid pMK12478 (GenBank: NC_022654.1) using the TPP software tool in TRANSIT (38). Insertion counts obtained from TPP were then analyzed in TRANSIT using the HMM to assign a most probable essential state call to each TA site and annotated gene.

### Comparative genomics

To identify mutually orthologous genes shared by *Mk* and *Mtb* H37Rv (GenBank: NC_000962.3), *MAH* MAH11 (GenBank: CP035744.1, aviumMD30 assembly; ASM312274v1), *Mab* ATCC 19977 (GenBank: CU458896.1), or *Msm* str. MC2 155 (GenBank: CP000480.1), GView Server was used to perform reciprocal BLAST searches between *Mk* and each species, with expected cutoff value, alignment length cutoff, and percent identity cutoff as 1.0E-10, 30, and 30, respectively (112). While more recent records are available for the *Msm* and *MAH* genomes, these particular accession numbers were used to facilitate downstream comparisons of essentiality between species. The orthology data along with the insertion counts from the aggregated TnSeq data from this study were visualized in the context of the *Mk* chromosome using the Gview application. The orthology data were cross-referenced with existing essentiality data available for each species (33 - 36). As noted in the Results section, the GView BLAST searches yielded results containing *Mtb* and *MAH* ORFs which were not present in corresponding essentiality data sets (34, 35). Closer inspection of annotated features of the corresponding genomes showed 18 such ORFs for *Mtb* and 54 such ORFs for *MAH* that are not present in the essentiality data sets. In the case of *Mtb*, the available TA site essentiality data (35) were used to assign an essentiality state call for each ORF (Data set S9). In the case of *MAH*, the essentiality state call of the ORFs were labeled N/D (not determined). The addition of these ORFs in our analysis explains why the total number of ORFs for these species differs from previous analyses. Venn diagrams were generated in the RStudio using the “Eulerr” package and then edited in Adobe Illustrator (Adobe, San Jose, CA, USA). For all *Mk* genes specifically discussed in the Results section, the TA site insertion and state call data (Data set S1) were scrutinized to identify local features (e.g., total number of TA sites, read counts, TA sites fitting the NP motif, CUTA segments) and ensure the reliability of the gene essentiality predictions.

## SUPPLEMENTAL MATERIAL

TABLE S1 mbio.00573-23-s0001.docx Sequencing statistics for the 12 replicate *M. kansasii* TnSeq data sets.

Dataset S1 mbio.00573-23-s0002.xlsx List of essentiality calls based on the motif-dependent Hidden Markov Model of essentiality for all TA sites and annotated genes in the *M*. *kansasii* ATCC 12478 chromosome.

Dataset S2 mbio.00573-23-s0003.xlsx *M. kansasii* ORFs and mutual orthologs identified in *M*. *tuberculosis*, *M*. *avium* subsp. *hominissuis*, *M*. *abscessus*, and *M*. *smegmatis* along with corresponding essentiality calls.

Dataset S3 mbio.00573-23-s0004.xlsx *M*. *kansasii*, *M*. *tuberculosis*, *M*. *avium* subsp. *hominissuis*, *M*. *abscessus*, and *M*. *smegmatis* ORF orthologs predicted to be essential.

Dataset S4 mbio.00573-23-s0005.xlsx Essentiality of *M*. *kansasii* non-ORF annotated genes.

Dataset S5 mbio.00573-23-s0006.xlsx *M*. *kansasii* intergenic regions containing four or more consecutive unoccupied TA sites (CUTA segment).

Dataset S6 mbio.00573-23-s0007.xlsx Essentiality calls based on the motif-dependent Hidden Markov Model of essentiality for all TA sites and annotated genes in the *M*. *kansasii* ATCC 12478 plasmid pMK12478.

Dataset S7 mbio.00573-23-s0008.xlsx *M*. *kansasii* pMK12478 and *M*. *avium* subsp. *hominissuis* pMD2 mutual orthologs along with essentiality state calls.

Dataset S8 mbio.00573-23-s0009.xlsx Essentiality of the five ESX loci in *M*. *kansasii* and *M*. *tuberculosis*.

Dataset S9 mbio.00573-23-s0010.xlsx Essentiality of *M*. *tuberculosis* genes not represented in Dejesus et al., 2017 (35).

ASM does not own the copyrights to Supplemental Material that may be linked to, or accessed through, an article. The authors have granted ASM a non-exclusive, world-wide license to publish the Supplemental Material files. Please contact the corresponding author directly for reuse.
