# Supplementary material for: Comprehensive essentiality analysis of the Mycobacterium kansasii genome by saturation transposon mutagenesis and deep sequencing
Source: mBio. 2023 Jun 23;14(4):e00573-23. doi: 10.1128/mbio.00573-23 (PMC10470612; doi:10.1128/mbio.00573-23)
Supplement: TABLE S1 — Sequencing statistics for the 12 replicate M. kansasii TnSeq data sets. [file mbio.00573-23-s0001.docx]

**Table S1. Sequencing statistics for the 12 replicate *M. kansasii* TnSeq datasets.**

| **Library ID** | **Read count^a^** | | | **Template count^b^** | | | **Number of TA sites hit^c^** | | | **Insertion density^d^** | | |
| --- | --- | --- | --- | --- | --- | --- | --- | --- | --- | --- | --- | --- |
|  | **Chromosome** | **Plasmid** | **Total** | **Chromosome** | **Plasmid** | **Total** | **Chromosome** | **Plasmid** | **Total** | **Chromosome** | **Plasmid** | **Total** |
| Mk 1 | 16,371,943 | 604,141 | 16,976,084 | 10,948,659 | 403,775 | 11,352,434 | 56,141 | 1,681 | 57,822 | 57.5% | 74.7% | 57.8% |
| Mk 2 | 6,576,423 | 251,147 | 6,827,570 | 3,576,969 | 136,310 | 3,713,279 | 49,225 | 1,527 | 50,752 | 50.4% | 67.8% | 50.8% |
| Mk 3 | 25,035,386 | 1,249,914 | 26,285,300 | 14,352,864 | 721,097 | 15,073,961 | 57,074 | 1,680 | 58,754 | 58.4% | 74.6% | 58.8% |
| Mk 4 | 23,345,009 | 1,062,835 | 24,407,844 | 13,403,236 | 614,682 | 14,017,918 | 64,110 | 1,835 | 65,945 | 65.6% | 81.5% | 66.0% |
| Mk 5 | 14,984,594 | 504,224 | 15,488,818 | 9,499,263 | 321,192 | 9,820,455 | 55,452 | 1,611 | 57,063 | 56.8% | 71.6% | 57.1% |
| Mk 6 | 20,004,053 | 935,226 | 20,939,279 | 7,353,307 | 343,060 | 7,696,367 | 43,378 | 1,409 | 44,787 | 44.4% | 62.6% | 44.8% |
| Mk 7 | 23,352,383 | 812,771 | 24,165,154 | 11,269,419 | 391,621 | 11,661,040 | 47,733 | 1,451 | 49,184 | 48.9% | 64.5% | 49.2% |
| Mk 8 | 15,716,650 | 633,880 | 16,350,530 | 9,854,794 | 396,865 | 10,251,659 | 53,386 | 1,613 | 54,999 | 54.6% | 71.7% | 55.0% |
| Mk 9 | 19,819,779 | 698,695 | 20,518,474 | 11,947,368 | 424,241 | 12,371,609 | 63,426 | 1,852 | 65,278 | 64.9% | 82.3% | 65.3% |
| Mk 10 | 18,090,299 | 802,416 | 18,892,715 | 11,360,492 | 508,197 | 11,868,689 | 53,306 | 1,666 | 54,972 | 54.6% | 74.0% | 55.0% |
| Mk 11 | 17,846,142 | 761,358 | 18,607,500 | 10,551,321 | 453,135 | 11,004,456 | 52,510 | 1,627 | 54,137 | 53.7% | 72.3% | 54.2% |
| Mk 12 | 21,608,844 | 865,034 | 22,473,878 | 12,621,462 | 508,943 | 13,130,405 | 65,087 | 1,877 | 66,964 | 66.6% | 83.4% | 67.0% |
| **Cumulative** | 222,751,505 | 9,181,641 | 231,933,146 | 126,739,154 | 5,223,118 | 131,962,272 | 84,803 | 2,175 | 86,978 | 86.8% | 96.6% | 87.0% |

^a^Total reads mapping to TA sites in the *M. kansasii* ATCC 12478 genome (chromosome [GenBank: NC_022663.1] and plasmid pMK12478 [GenBank: NC_022654.1]). ^b^Reduction of mapped reads to unique templates using barcodes (random barcodes introduced to fragments during library preparation). ^c^Number of TA sites with at least one insertion. ^d^Percent TA sites hit (chromosome: 97,702 total TA sites; plasmid: 2,251 total TA sites).
